# The effects of exercise training on maximum aerobic capacity, resting heart rate, blood pressure and anthropometric variables of postmenopausal women with breast cancer

**Published:** 2010

**Authors:** Nader Rahnama, Reza Nouri, Farhad Rahmaninia, Arsalan Damirchi, Hamid Emami

**Affiliations:** a Associate Professor, Faculty of Physical Education and Sport Sciences, University of Isfahan, Isfahan, Iran; b PhD Student, Exercise Physiology, Faculty of Physical Education and Sport Sciences, University of Guilan, Rasht, Iran; c Professor, Faculty of Physical Education and Sport Sciences, University of Guilan, Rasht, Iran; d Assistant Professor, Faculty of Physical Education and Sport Sciences, University of Guilan, Rasht, Iran; e Assistant Professor, Department of Radiotherapy, School of Medicine, Isfahan University of Medical Sciences, Isfahan, Iran

**Keywords:** Breast Cancer, Postmenopausal, Maximum Aerobic Capacity, Blood Pressure, Anthropometric Variables

## Abstract

**BACKGROUND:**

The aim of this study was to investigate the effects of exercise training on maximum aerobic capacity, resting heart rate (RHR), blood pressure and anthropometric variables of postmenopausal women with breast cancer.

**METHODS:**

Twenty nine women with breast cancer who received surgery, chemotherapy and radiotherapy with current hormone therapy were divided into two groups; intervention and control. Subjects in the intervention group performed 15 weeks combination exercise training including walking for 25 to 45 minutes (2 sessions per week) and resistance training for 60 minutes (2 sessions per week that were different from walking days). In pre and post tests, VO_2_max, RHR, blood pressure, body weight, body mass index (BMI) and waist to hip ratio (WHR) were measured in both groups. Data was analyzed using analysis of covariance (ANCOVA).

**RESULTS:**

Significant differences were observed for VO _2_max, RHR, body weight, BMI and WHR between intervention and control groups after 15 weeks (p < 0.05). In fact, exercise training had positive effects on the VO _2_max, RHR, body weight, BMI and WHR in postmenopausal women with breast cancer. No significant different was found for blood pressure between two groups (p > 0.05).

**CONCLUSIONS:**

It can be concluded that exercise training may improve maximum aerobic capacity, RHR and anthropometric variables in postmenopausal women with breast cancer.

After diagnosis and treatment of breast cancer, the functional capacities of women with breast cancer significantly decline and remain low for a long time after treatment.^1^ So, it is not surprising that the researchers have reported decreases in the cardiorespiratory functions after cancer, especially breast cancer.^2^ Also, it has been indicated that treatment of breast cancer has side effects on the mental and physical aspects.^1^ Some of these effects are exhaustion resulting from surgery, chemotherapy and radiotherapy, decreased aerobic capacity, weight gain (between 2 to 6 kg) and hypertension.^3^

It is obvious that the maximum oxygen consumption or VO_2_max is an important factor in the determining of the cardiorespiratory function or aerobic capacity; VO_2_max is the maximum oxygen that one can utilize during a maximum activity.^4^ Previous research showed that exhaustion resulting from treatment of breast cancer reduces the aerobic capacity in women with breast cancer, significantly.^2^ Also, some reports indicated that breast cancer decreases VO_2_max of women.^2^,^5^–^7^ Therefore, some studies have evaluated the effects of aerobic exercise training on VO_2_max in women with breast cancer. The findings of these studies showed that exercise training can improve VO_2_max in breast cancer survivors.^2^,^5^–^9^ For instance, McNeely et al (2006) demonstrated that physical activity improves the VO_2_max in women with breast cancer up to 12%, on average.^10^ It seems that cancer and its treatments reduce VO_2_max in postmenopausal women with breast cancer. Perhaps, exercise training can improve VO_2_max in these patients.

Surgery, chemotherapy and radiotherapy are current treatment for breast cancer patients.^11^ Wilson et al (2005) showed that chemotherapy and radiotherapy had a side effect on the blood pressure and causes hypertension.^12^ Recently some investigators have studied the effect of exercise training on the blood pressure of the women with breast cancer. They showed exercise training can reduce blood pressure in women with breast cancer.^9^,^12^–^14^ It seems that exercise, specially walking can reduce the blood pressure in the women with breast cancer.

Body weight, body mass index (BMI) and the ratio of waist to hip (WHR) are important anthropometric variables. High BMI and overweight are important factors that can increase the risk of breast cancer and possibility of its recurrence.^15^ In fact, overweight and high BMI increase the risk of breast cancer in the postmenopausal woman.^16^ Also Malin et al demonstrated that high BMI and inactive lifestyle can increase risk of breast cancer in postmenopausal women.^17^ Therefore, some researchers are interested to study the effect of exercise training on the anthropometric variables of women with breast cancer during and after treatment. Findings of Wilson et al (2005) indicated that exercise training reduce BMI, body weight and fat percentage of women with breast cancer.^12^ However, McNeely et al and Matthews et al (2007) showed that there was no significant change in weight, BMI and body composition of women with breast cancer.^10^,^18^ Schmitz et al (2005) and Ohira et al (2006) demonstrated that resistance exercise training can improve the body composition of women with breast cancer.^9^,^19^ It is possible that exercise training can reduce the body weight and BMI in postmenopausal women.

Although, there are some reports about the aerobic capacity, blood pressure and anthropometric variables as a result of exercise training in women with breast cancer, however these important variables have not well been documented. So, the aim of this study was to evaluate the effects of exercise training on maximum aerobic capacity, RHR, blood pressure and anthropometric variables of postmenopausal women with breast cancer.

## Methods

### 

#### Subjects

Subjects selected from the Center of Oncology and Radiotherapy of Hazrate Seyodoshohada Hospital in Isfahan. A list of the names and medical records of 1341 women with breast cancer who came to this center for treatment from 2005 to 2008 were given a survey. After primary survey, 342 women of 50 to 65 years old were selected. These women received surgery, chemotherapy and radiotherapy and they had current hormone therapy. They were in stage I to IIIB. They should not have specific illness and in the past 6 month should not have any experience of a menstrual cycle. Also, they should not have participated in any exercise training or physical activity in past 6 month and their body weight should not have changed in this period (last 6 month) as much as 10% of their whole body weight. All the patients invited to participate in this study by telephone. Among all, 58 women indicated their readiness to participate in this study and 38 women were present in meeting day. The physical activity readiness questionnaire (PAR-Q) and written informed consent was obtained from all of the subjects. By surveying the questionnaire, it was specified that 32 of them had conditions for taking part in this study. These 32 women divided into two groups randomly; intervention group and control group. At the end of exercise training program 29 women (15 women in control group and 14 women in intervention group) completed the relevant measurements of the post tests. This study was approved by the Faculty of Physical Education and Sport Sciences of the University of Guilan.

#### Measurements

Subjects’ height was measured by stadiometer (Seca model, made in Germany). Body weight of subjects was measured without shoes by a digital scale (Pand Electronic model, made in Iran). BMI was obtained by dividing the weight in kilogram to square of the height in meter. Waist and hip circumference was obtained by band tape in meter. WHR was calculated by dividing the waist circumference to hip circumference. Blood pressure was measured with a Japanese sphygmomanometer model ALPK2 in seated position. Since any activity can increase HR it was measured immediately after waking up. Also, HR is in lowest rate in the morning, immediately after waking up. Therefore, the RHR was recorded using the heart rate monitor belt (Polar model) by the subjects immediately after waking up. For measuring the VO_2_max in two groups modified Bruce protocol was used. Bruce protocol is one of the most common methods for estimating VO_2_max. This test performed on a treadmill. The standard Bruce test was started at 2.74 km/h (1.7 mph) and at incline of 10% for 3 minutes. At three minutes intervals the incline of treadmill increased by 2% and speed increase to 4.02, 5.47, 6.76, 8.05, 8.85, 9.65, 10.46, 11.26 and 12.07 km/h in each stage (10 stages in total), respectively. The modified Bruce protocol started at 2.74 km/h and incline of 0% and 2.74 km/h and 5% incline and third stages corresponding to the first stage of the standard Bruce test. The time taken on the treadmill recorded in minutes (T). The VO_2_max formula for women is :

VO_2_max = 4.38 × T - 3.9

All the measurements were obtained twice and recorded by one staff that was blinded to subjects in pre and post tests.

#### Exercise Training Protocol

This protocol was only designed for the intervention group. This group performed 60 minute resistance training twice weekly for 15 weeks at the Foolad Mobarake Sepahan Sport Club. They performed three sets each of nine common resistance training exercises; subjects lifted as much weight as they could for 10 repetitions per set from 1^st^ to 5^th^ week, 12 repetitions per set from 6^th^ to 10^th^ week and 14 repetitions per set from 11^th^ to 15^th^ week. The nine resistance training exercises included exercises performed on Cybex strength training equipment (Smith press squats, leg press, leg extension, seated leg curl, lat pulldowns) and with free weights (bench press, overhead press, biceps curls, and triceps kickbacks). Indeed, these subjects took part in supervised walking program for two times per week, differed from resistance training days. The walking program started at 45% of maximum heart rate for 25 minutes in weeks 1-5. Duration of walking from 6^th^ -10^th^ weeks was 35 minutes and intensity was 55% of maximum heart rate. From 11^th^ -15^th^ weeks, duration of walking was 45 minutes with intensity of 65% of maximum heart rate. Heart rate controlled by Polar heart rate belt. The control group participated in measurements only and they were asked not to participate in any physical activity or exercise training.

#### Statistical Analysis

Data were analyzed using SPSS software (Version 13.0). For the description of data, mean and standard deviation were used. Also, mean values of two groups in pre and post tests were compared by analysis of covariance (ANCOVA) for VO_2_max, RHR, blood pressure, body weight, BMI and WHR. The significance level of this study was set at p < 0.05.

## Results

Table 1 shows the VO_2_max, RHR, blood pressure, body weight, BMI, waist circumference, hip circumference and WHR values of two groups in pre and post tests. In the intervention group, mean of VO_2_max (from 17.5 to 20.65 ml.kg^-1^. min^-1^) increased and means of RHR (from 88.57 to 83.92), blood pressure (from 124.33 to 123.61 mmHg), body weight (from 70.39 to 69.40 kg), BMI (from 28.04 to 27.74 kg/m^2^) and WHR (from 0.982 to 0.956 m) decreased after 15 weeks of exercise training. There were significant differences between (two) groups in VO_2_max (F = 11.24, p = 0.002), RHR (F = 6.15, p = 0.000), body weight (F = 5.22, p = 0.031), BMI (F = 5.94, p = 0.022) and WHR (F = 9.24, p = 0.001) at post tests. However, there was no significant difference between groups in blood pressure (F = 1.21, p = 0.11) at post tests. Findings showed that exercise training had significant effect on the VO_2_max, RHR, body weight, BMI and WHR in the intervention group after 15 weeks.

**Table 1:** The values of VO_2max_, RHR, blood pressure and anthropometric variables in intervention and control groups in pre and post test

Groups	Control group		Intervention group	
Variables	Pre test	Post test	Pre test	Post test
VO_2_max (ml/min/kg)	14.45 ± 5.05	13.85 ± 5.18	17.15 ± 6.02	20.65 ± 5.73
RHR (beat/min)	88.53 ± 6.30	89.46 ± 5.94	88.57 ± 5.35	83.92 ± 5.62
Blood pressure (mmHg)	127.45 ± 4.90	129.13 ± 5.23	124.33 ± 6.36	123.61 ± 3.79
Body weight (kg)	70.170 ± 9.00	71.60 ± 9.28	70.39 ± 12.75	69.40 ± 13.51
BMI (kg/m^2^)	27.42 ± 3.43	27.98 ± 3.55	28.04 ± 4.69	27.74 ± 4.77
Waist circumference (m)	0.93 ± 0.08	0.93 ± 0.08	0.99 ± 0.1	0.96 ± 0.12
Hip circumference (m)	0.97 ± 0.09	0.97 ± 0.09	1.1 ± 0.1	1.1 ± 0.09
WHR (m)	0.96 ± 0.03	0.96 ± 0.04	0.98 ± 0.07	0.94 ± 0.07

## Discussion

The aim of this study was to investigate the effects of exercise training on maximum aerobic capacity, RHR, blood pressure and anthropometric variables of postmenopausal women with breast cancer.

### 

#### Maximum Aerobic Capacity

The results of this study showed that the maximum aerobic capacity of intervention group was increased by 3.49 ml.min^-1^. kg^-1^ and maximum aerobic capacity of the control group was decreased by 0.7 ml.min^-1^.kg^-1^ at post tests.

Also, it demonstrated that combination of resistance and aerobic exercise training for 15 weeks had significant effect on the aerobic capacity of the postmenopausal women with breast cancer. In Visovski et al (2005) study functional and aerobic capacity was improved in postmenopausal women with breast cancer after 12 weeks.^7^ Mustian et al (2006) reported that combination exercise training can improve aerobic capacity of the women with breast cancer.^2^ Schneider et al (2007) reported that mid intensity controlled exercise training maintains or improves the aerobic capacity.^9^ Also, Nikander et al (2007) and Mc Neely et al (2006) showed that exercise training can maintain or improve the aerobic capacity in women with breast cancer.^6^,^10^ The findings of present study confirm the previous studies and shows that exercise training can improve the VO_2_max in the postmenopausal woman with breast cancer. Although the aerobic capacity of women decreases as a result of breast cancer and its treatments, but exercise training may improve the aerobic capacity. It seems that the improvement in the maximum aerobic capacity as a result of exercise training could cause an improvement in the functional capacity of the postmenopausal woman with breast cancer. Also, improvement in the maximum aerobic capacity may decrease the signs of exhaustion resulting from the treatment in postmeno-pausal women with breast cancer.

#### Resting Heart Rate (RHR)

In this study, RHR of the intervention group was decreased by 4.65 beat/min and RHR of the control group was increased by 0.93 beat/min. There was a significant difference between RHR of two groups, after 15 weeks. Also, this finding shows that the resistance and aerobic exercise training can have a significant effect on the decrease of RHR in postmenopausal women with breast cancer. This finding is in agreement with others.^2^,^6^,^20^ Therefore, it seems that combination exercise training can decrease RHR in the postmenopausal women with breast cancer. The mechanism for reduction of RHR can be due to improvement of cardiovascular system.

#### Blood Pressure

In the present study exercise training did not have a significant effect on the decrease of blood pressure of the postmenopausal women with breast cancer. Nevertheless, after 15 weeks blood pressure had a 0.72 mmHg decrease in the intervention group and 1.68 mmHg increases in the control group. This finding is not in agreement with others.^9^,^12^–^14^ The contradiction of this result and the previous study may be related to the nature of the exercise training. It seems that moderate aerobic exercise training such as walking have better effects on the decrease of blood pressure than the combination or resistance training. Therefore, it can be possible that combination exercise training may not be improving the blood pressure of the postmenopausal women with breast cancer.

#### Anthropometric Variables

The findings of this study indicated that exercise training has significant effects on body weight loss and BMI of the intervention group. After 15 weeks, body weight and BMI of the intervention group decreased by 0.99 kg and 0.3 kg/m^2^, respectively. In the control group after 15 weeks, body weight and BMI increased by 1.43 kg and 0.54 kg/m^2^, respectively. Also, the findings of this study showed that combination exercise training has a significant effect on WHR of postmenopausal women with breast cancer. This finding is in agreement with Wilson et al (2005) and Ohira et al (2006) studies.^12^,^19^ However, this finding is not in agreement with McNeely et al (2006) and Matthews et al (2007) studies that reported the absence of significant effect of exercise training on body weight, BMI and WHR of women with breast cancer.^10^,^18^ The possible reason for this can be related to the type and duration of exercise training. Because Matthews et al (2007) used uncontrolled walking program for 12 weeks,^18^ while in the present study supervised combination exercise training was used for 15 weeks. Also, McNeely et al (2006) studied the effect of exercise training on all women with breast cancer at any age with any treatment.^10^ Whereas, the present study was limited to postmenopausal women with breast cancer who was between 50 to 65 years old and have had surgery, chemotherapy and radiotherapy treatments and had current hormone therapy. It seems that for the effectiveness of the exercise training on body weight, BMI and WHR of women with breast cancer, researchers should use supervised exercise training program.

## Conclusions

In conclusion, supervised exercise training may have positive effects on the maximum aerobic capacity, RHR and anthropometric variables of postmenopausal women with breast cancer. However, exercise training does not have a significant effect on blood pressure of postmenopausal women with breast cancer. Therefore, exercise training has positive effects for postmenopausal women with breast cancer, however, these findings need to be confirmed in future studies.
